# Comment on: Endogenous Ouabain and Related Genes in the Translation from Hypertension to Renal Diseases, *Int. J. Mol. Sci.* 2018, *19*, 1948

**DOI:** 10.3390/ijms20030505

**Published:** 2019-01-24

**Authors:** Hauke Fürstenwerth

**Affiliations:** Unterölbach 3A, 51381 Leverkusen, Germany; hauke@fuerstenwerth.com; Tel.: +49-2171-733740; Fax: +49-2171-733750

**Keywords:** endogenous ouabain, hypertension, heart failure

Dear Editor,

In their review “Endogenous Ouabain and Related Genes in the Translation from Hypertension to Renal Diseases” [1], Paolo Manunta and coworkers describe a potentially pathogenic role of endogenous ouabain (EO) in various diseases, including renal failure, essential hypertension and heart failure. They assert that EO has significant implications in the pathogenesis of many common diseases. The authors suggest that EO acts as a pro-hypertrophic and growth-promoting hormone, which might lead to cardiac remodeling affecting cardiovascular function and structure. In addition, a possible role of EO in the development of acute kidney injury is hypothesized.

Although it is claimed that EO is identical to plant-derived ouabain, no reference is given to the more than one hundred years of clinical experience with ouabain in the treatment of heart diseases. Ouabain and the related *Strophanthus* glycoside, k-Strophanthin, by default have been used for more than a century to treat heart diseases. The structurally similar drugs, cymarin and convallatoxin, have also been used [2]. Ouabain is found in both *Acokanthera ouabaio* and *Strophanthus gratus*. The glycoside occurring in *Strophanthus kombé* is k-Strophanthin. Herman Thoms isolated the pure glycosides from S. kombé and S. gratus in 1904 and has unambiguously assigned them with the names k- and g-Strophanthin [3], the latter is referred to as “ouabain” in the English literature. Based on the work of Thomas Fraser, Burroughs, Wellcome & Co in 1886 introduced a *S. kombé* extract called “Tincture of *Strophanthus*”. This was sold at seven shillings per ounce. In America, E.R. Squibb and Sons was one of the first suppliers of *Strophanthus* preparations. Particularly popular was a chocolate-coated tablet of a mixture of Digitalis and *Strophanthus* extracts, which was sold at 16 cents per one hundred pieces [4]. In 1889, Boehringer Mannheim introduced pure k-Strophanthin to the market. In cooperation with Albert Fraenkel Boehringer, in 1907 introduced a solution of k-Strophanthin for intravenous administration under the brand name “Kombetin”.

In 1904, E. Merck, Darmstadt, commercialized a standardized solution of pure ouabain as “g-Strophanthin crystallisatum nach Thoms”. In 1906, Kali-Chemie also began marketing an ouabain solution under the trade name “Purostrophan”. In 1909, the French physician Henri Vaquez introduced the intravenous application of ouabain (“Ouabain-Arnaud”) in France. In World War I medical personnel in the German army, by order of the ambulance corps, exclusively used ouabain solutions to treat heart failure [5]. The therapeutic profile and the disease profiles for which the use of *Strophanthus* glycosides is appropriate are documented in many reports on clinical experiences and have been summarized in numerous reviews, mostly in the German literature [6]. As early as the first half of the 20th century distinguished scientists such as Albert Fraenkel, University of Heidelberg, and Ernst Edens, University of Dusseldorf, published monographs [7,8] that document in detail the clinical effects of *Strophanthus* glycosides. In textbooks ouabain has been praised as “*the biggest advance in cardiac therapy since Withering in 1785*” [9].

Ouabain-based products, over the decades of their use, have been given as standard medication to millions of heart failure patients. The database of the German Institute for Medical Documentation and Information records more than 20 orally administered ouabain preparations that were used in Germany after 1950. Decades of clinical experience with ouabain provide a yardstick by which all research results and hypotheses related to ouabain have to be measured. Observations at the bedside are more meaningful than speculative hypotheses based on experimental research. It is totally incomprehensible why Manunta et al. do not mention the well-documented clinical experiences with ouabain in their review. It is also incomprehensible that current reports on cardioprotective effects of ouabain are not mentioned either. Current research findings indicate cardio protection is induced by ouabain [10,11,12]. Lijune Liu and co-workers report that ouabain is beneficial to various stages of heart failure [13]. None of these reports are mentioned by Manunta et al.

Based on historical clinical experiences and current research findings, it can be ruled out that ouabain, that has been used successfully for decades in the treatment of heart failure, will cause heart failure. Equally excluded is that ouabain produces hypertension. In clinical practice ouabain lowers blood pressure [14]. Already Fraser had pointed out that “*strophanthin increases the action of the heart without raising blood pressure.*”

Although the authors postulate that EO damages the kidney, current findings on renal protective effects of ouabain are not mentioned. Aperia et al. report that ouabain prevents adverse programming of kidney development from negative effects of malnutrition [15,16]. Ouabain (0.1–10 nM) also was found to stimulate proliferation and increase the viability of kidney cells [17].

Based on all available data it can be ascertained that the mutually exclusive effects of plant derived ouabain and the inhibitor of the Na/K-ATPase observed in mammalian tissues that reacts with ouabain based antibodies, do not support the hypothesis that this inhibitor is identical with ouabain, but favor the interpretation that “endogenous ouabain” is something different.

This conclusion is supported by current analytical findings. Vogeser et al. established a stable-isotope dilution API-MS/MS method for the quantification of ouabain in human plasma [18]. This team developed a method of extremely high sensitivity for detecting spiked ouabain in human plasma. The method was fully validated according to FDA guidelines and published in the official *Journal of the International Federation of Clinical Chemistry* after peer review. Using this method, no ouabain could be detected in unspiked human plasma samples that contained EO levels of 206–665 pmol/l as determined by radioimmunoassays in the laboratory of Paolo Manunta [19].

While some research groups have identified endogenous ouabain in human plasma, others have failed to detect EO [20]. There was a lack of attempts to determine EO in identical samples using different methods by different laboratories. This essential gap has been closed by Vogeser’s experiments. While no EO could be detected by using API-MS/MS, radioimmunoassays performed by Manunta in his laboratory on the same samples indicated EO in substantial concentrations. These findings exemplify that antibody-based radioimmunoassays are often subject to cross reactivity with compounds other than those to which the antibody was raised. Another current example is ionotropin. This substance has been isolated from mammalian tissue. It cross reacts with digoxin-specific antibodies, but has a proposed chemical structure that is not related to digoxin [21]. This inherent methodological disadvantage also applies to the work of Takahashi and coworkers [22]. Their detection of endogenous ouabain, too, is based on a sensitive enzyme-linked immunosorbent assay for ouabain. Based on his findings that endogenous ouabain binds to plasma proteins, Takahashi, in his purification process, uses an acidification step to liberate endogenous ouabain. Contrary to these findings it is well known that ouabain, unlike digitalis glycosides, does not bind to plasma proteins [23]. This fact also confirms that endogenous ouabain is different from ouabain. From LC/MS measurements, Takahashi et al. concluded that the endogenous ouabain “molecule may be one of the isomers of ouabain, and the molecular size is the same as authentic plant-derived ouabain.” This hypothesis has not been verified by proven analytical methods such as API-MS/MS.

The results of Vogeser’s team are unequivocal: There is no ouabain in human plasma. “Endogenous ouabain” is different from ouabain. Decades of clinical experience with ouabain and current analytical findings refute the hypothesis of “endogenous ouabain”. Time has come to concentrate again on the therapeutic effects of ouabain.

The hypothesis of the existence of “endogenous ouabain” has been subject to a fierce scientific debate [24]. Arguments and findings of scientific experiments must be judged with self-criticism. Science is a set of methods aimed at building a testable body of knowledge open to rejection or confirmation. Therefore, in the end, truth will prevail. For the locals in Africa, *Strophanthus* was a poison and remedy in one. In the mythology of the tribe of the Wilé in Upper Volta, this plant was sent from paradise to the earth to heal or punish people according to their merit [25]. A clinical reassessment of ouabain offers a unique opportunity to transform this gift from paradise into much needed new treatment options for cardiovascular diseases.
